# Neuronal activity induces symmetry breaking in neurodegenerative disease spreading

**DOI:** 10.1007/s00285-024-02103-x

**Published:** 2024-05-13

**Authors:** Christoffer G. Alexandersen, Alain Goriely, Christian Bick

**Affiliations:** 1 Mathematical Institute, University of Oxford, Oxford, UK; 2 Department of Mathematics, Vrije Universiteit Amsterdam, Amsterdam, The Netherlands; 3 Amsterdam Neuroscience – Systems and Network Neuroscience, Amsterdam, The Netherlands

**Keywords:** Neurodegenerative disease, Human connectome, Dynamics on networks, Brain activity, Toxic spreading, Adaptive networks, Multiple time scales, Alzheimer’s disease, 37N25, 62P10, 92C20

## Abstract

Dynamical systems on networks typically involve several dynamical processes evolving at different timescales. For instance, in Alzheimer’s disease, the spread of toxic protein throughout the brain not only disrupts neuronal activity but is also influenced by neuronal activity itself, establishing a feedback loop between the fast neuronal activity and the slow protein spreading. Motivated by the case of Alzheimer’s disease, we study the multiple-timescale dynamics of a heterodimer spreading process on an adaptive network of Kuramoto oscillators. Using a minimal two-node model, we establish that heterogeneous oscillatory activity facilitates toxic outbreaks and induces symmetry breaking in the spreading patterns. We then extend the model formulation to larger networks and perform numerical simulations of the slow-fast dynamics on common network motifs and on the brain connectome. The simulations corroborate the findings from the minimal model, underscoring the significance of multiple-timescale dynamics in the modeling of neurodegenerative diseases.

## Introduction

Mathematical models of dynamical processes on networks are crucial to our understanding of pandemics, economics, opinion formation, evolution, ecology, and neurodegenerative disease (Siettos and Russo 2013; Schweitzer et al. 2009; Ureña et al. 2019; Proulx et al. 2005; Lloret-Villas et al. 2017). While the underlying network structure is traditionally assumed to be static, it has become clear that network adaptivity is a crucial constituent to many real-world dynamical systems (Gross and Blasius 2007; Berner et al. 2023). For example, infectious diseases spreading (Gross et al. 2006) and biological neural circuits (Butz et al. 2009) alter the network structures they are evolving on, establishing a feedback loop between dynamics and network structure. Networks exhibiting such mutual interactions between dynamical processes and network topology are referred to as adaptive, or coevolutionary, networks (Maslennikov and Nekorkin 2017; Wang et al. 2019; Gross and Blasius 2007). In a range of applications, including oscillator networks (Gkogkas et al. 2022; Jüttner and Martens 2023; Ratas et al. 2021; Thiele et al. 2023), consensus dynamics (Jardón-Kojakhmetov and Kuehn 2020), and epidemic-resource dynamics (Böttcher et al. 2015), coevolutionary dynamics may also operate on disparate timescales. Although techniques from geometric singular perturbation theory (Kuehn 2015) and averaging theory (Sander et al. 2007) can provide insights into the emerging multiple-timescale dynamics, these methods become daunting in high dimensions.

A crucial—yet poorly understood—example of an adaptive network with multiple timescale dynamics is the human brain during neurodegenerative diseases, such as Alzheimer’s disease. The defining feature of Alzheimer’s disease is the accumulation of toxic variants of amyloid-$\beta$ and tau protein aggregates throughout the brain (Duyckaerts et al. 2019). It is believed that these toxic variants are produced by a prion-like mechanism, where toxic variants of the protein transform healthy variants into toxic ones (Harris et al. 2020). Although both amyloid-$\beta$ and tau are fundamental to the disease, the presence of tau correlates more significantly with cognitive decline. Furthermore, it has been shown that tau proteins spread throughout the brain following axonal pathways (Vogel et al. 2020; Cho et al. 2016), leading to neurodegeneration and decreases in neuronal activity levels (Harris et al. 2020). Tau proteins tend to follow a general spreading sequence—called the Braak staging pattern—starting in the entorhinal cortex. However, the basis for the initiation of Braak staging in the entorhinal cortex and the ensuing spreading pattern remains disputed. Furthermore, subgroupings of patients according to systematic aberrations in Braak staging patterns further complicate our picture of the disease (Duits et al. 2021; Ferreira et al. 2020). In recent years, however, it has become clear that neuronal activity plays a crucial role in the spreading of tau protein. Specifically, it has been shown that neurons with higher firing rates transport tau proteins at a higher rate into their neighbors (Wu et al. 2016; Sokolow et al. 2015; Pooler et al. 2013). As such, neuronal activity increases the outward transport of tau proteins, while tau proteins lower neuronal activity levels. Importantly, protein spreading and neuronal activity evolve on vastly different timescales; protein spreading operates on a timescale of years while neuronal activity operates on a timescale of seconds. The newly discovered bidirectional relationship between neuronal activity and protein spreading may be the missing link in our understanding of Alzheimer’s disease and other neurodegenerative diseases.

Mathematical modeling of neurodegenerative diseases has mostly focused on protein spreading and neuronal activity in isolation. On the one hand, the slow evolution of the protein spreading and subsequent damage to the neural networks in Alzheimer’s disease can be captured with a continuum approach using Fisher-KPP equations (Weickenmeier et al. 2018). However, protein spreading dynamics can also be effectively modeled from a network perspective using structural network reconstructions of the human brain. This idea was initially introduced in Raj et al. (2012)—and later expanded in Fornari et al. (2019)—and involves simplifying continuum models, easing the investigation of staging patterns (Putra et al. 2021, 2023) and parameter estimation through Bayesian techniques (Schäfer et al. 2022). Furthermore, a separate and successful approach employs a heterodimer model to investigate how amyloid-$\beta$ and tau spread during Alzheimer’s disease (Thompson et al. 2020; Brennan et al. 2023). On the other hand, the activity of individual neurons that give rise to neural oscillations—which are fast relative to disease progression—are captured by models of neuronal dynamics, such as the highly detailed Hodgkin–Huxley model which can emulate pathologies by incorporating defects in ion channel conductivity. However, the Hodgkin–Huxley model becomes intractable in larger networks of neurons, where oscillator models such as Kuramoto oscillators (Kuramoto 1975), theta neurons (Ermentrout and Kopell 1986), and integrate-and-fire neurons (Burkitt 2006) have shown great utility. In these models, the instantaneous oscillator frequencies are commonly interpreted as neuronal firing rates, which is a common metric for neuronal activity. Only recently have the two aspects of slow disease progression and fast neural dynamics been captured in a single modeling framework; see for example (Goriely et al. 2020; Alexandersen et al. 2023).

Motivated by the progression of Alzheimer’s disease, we here develop a multiple timescale approach to elucidate the dynamics of spreading processes and oscillator dynamics on adaptive networks. More specifically, we formulate a multiple timescale system where a slow heterodimer spreading process occurs on a network of fast Kuramoto oscillators. The presence of protein slows the natural frequencies of the Kuramoto oscillators, while the instantaneous frequencies of the Kuramoto oscillators increase the outward transport of protein from their respective nodes. The network structure is adaptive, as the Kuramoto frequencies alter the transport rates by scaling the link weights of the spreading network. In other words, the Kuramoto oscillators are enforcing a global adaptivity rule on the spreading process. With the goal of elucidating the role of fast oscillatory processes on the spreading patterns and vice versa, we begin by studying a minimal two-node model using slow manifold reduction and ad hoc averaging before corroborating our findings with numerical simulations of the generalized network model. We find that heterogeneously distributed frequencies of oscillators destabilize the spreading process by lowering the threshold for toxic outbreaks and inducing symmetry breaking in the spreading patterns. Moreover, we find two modes for toxic outbreaks: conversion-dominated and shunting-dominated spreading.

This article is organized as follows: In Sect. 2, we consider the heterodimer model on a minimal network of two nodes with asymmetric link weights to reflect the effect of activity on the spreading dynamics. In Sect. 3, we consider a multiple timescale two-node system, now equipped with both heterodimer and Kuramoto dynamics, which we call the *heterodimer-oscillator*. In Sect. 4, we support the findings from the minimal heterodimer-oscillator system by performing numerical simulations on common motifs found in complex networks and investigating the effect oscillatory activity can have on tau spreading in the human brain during Alzheimer’s disease.

## Heterodimer dynamics

In this section, we build on the classical heterodimer model for a simple 2-node graph and introduce asymmetry in the coupling between the nodes to understand its impact on the system dynamics. Specifically, we identify a pair of fixed points exchanging stability at a transcritical bifurcation and observe that the asymmetrical coupling not only shifts the location of this bifurcation in parameter space but also disrupts symmetries within the fixed points. The dynamical behavior of the asymmetrically-coupled heterodimer model will be instrumental in our analysis of the full system with coevolutionary spreading and oscillator dynamics later on in Sect. 3.

### The heterodimer model

The heterodimer model describes a process of healthy proteins being converted into toxic proteins by a second-order rate equation. The heterodimer model is often used in the context of networks, over which both the healthy and toxic proteins are spreading. We assume that the process takes place on a network with *N* nodes defined by a weighted adjacency matrix ${\textbf{W}} = (W_{ij})$. For ${\textbf{W}}$ we define the standard graph Laplacian ${\textbf{L}}= (L_{ij})$ with components

1 $$\begin{aligned} L_{ij}=-W_{ij}+\delta _{ij}\sum _{j=1}^{N} W_{ij}, \end{aligned}$$

where $\delta _{ij}$ is the Kronecker symbol. According to the heterodimer model, the evolution of the concentration of healthy proteins $u_i \ge 0$ and of toxic proteins $v_i \ge 0$ at node *i* is given by

2a $$\begin{aligned} \dot{u}_i&= -\sum _{j=1}^N L_{ij} u_j + k_0 - k_1 u_i - k_2 u_i v_i, \qquad i=1,\ldots ,N, \end{aligned}$$

2b $$\begin{aligned} \dot{v}_i&= -\sum _{j=1}^N L_{ij} v_j - k_3 v_i + k_2 u_i v_i, \ \ \qquad \qquad i=1,\ldots ,N, \end{aligned}$$

where $k_0 > 0$ is the healthy protein production rate, $k_1 > 0$ and $k_3 > 0$ are the healthy and toxic clearance (protein degradation) rates, and $k_2 > 0$ is the rate of conversion from healthy to toxic proteins.

**Fig. 1:**
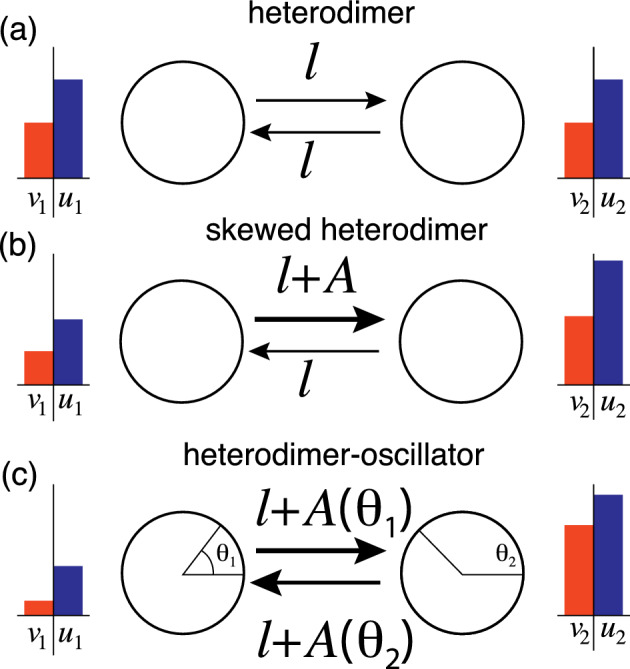
Overview of the heterodimer variations. **a** The original heterodimer model, with healthy and toxic species transported between the nodes at equal rates. **b** The skewed heterodimer model where node 1 has higher activity and thus increases the transport rate into node 2. Note that toxic species do not affect the activity parameter *A*. **c** The heterodimer-oscillator model, where each node harbors an oscillator operating at a faster time rate than the spreading process. The oscillators are coupled and their frequency determines the transport rate of species between the nodes; in this illustration, node 1 has a higher frequency. Conversely, the toxic species affect the intrinsic frequency of the oscillators

With the ultimate goal of understanding how the possible dynamics of this system are affected by oscillatory activity, we start with the simple case of two nodes connected by an undirected link as shown in Fig. 1a:

3a $$\begin{aligned} \dot{u}_1&= - \ell u_1 + \ell u_2 + k_0 - k_1 u_1 - k_2 u_1 v_1, \end{aligned}$$

3b $$\begin{aligned} \dot{v}_1&= -\ell v_1 + \ell v_2 - k_3 v_1 + k_2 u_1 v_1, \end{aligned}$$

3c $$\begin{aligned} \dot{u}_2&= \ell u_1 - \ell u_2 + k_0 - k_1 u_2 - k_2 u_2 v_2, \end{aligned}$$

3d $$\begin{aligned} \dot{v}_2&= \ell v_1 - \ell v_2 - k_3 v_2 + k_2 u_2 v_2, \end{aligned}$$

where $\ell > 0$ is the single, reciprocal weight link. Note that all parameters and variables are nonnegative. The system has two fixed points. In general, we refer to a fixed point as *healthy* if $v_i=0$ for all $i=1,\ldots ,N$ and *toxic* if $v_i>0$ for at least one $i\in \{1,\ldots ,N\}$. The 2-node heterodimer model has exactly one healthy fixed point (denoted by a superscript $\textrm{H}$) and one toxic fixed point (superscript $\textrm{T}$), given by

4a $$\begin{aligned}&u_1^\textrm{H} = u_2^\textrm{H} = \frac{k_0}{k_1},\quad v_1^\textrm{H} = v_2^\textrm{H} = 0, \end{aligned}$$

4b $$\begin{aligned}&u_1^\textrm{T} = u_2^\textrm{T} = \frac{k_3}{k_2}, \quad v_1^\textrm{T} = v_2^\textrm{T} = \frac{\kappa }{k_2 k_3}, \end{aligned}$$

where $$\kappa = k_0 k_2 - k_1 k_3$$.

In terms of the dynamics, we are mostly interested in the transition between healthy states and toxic states. In other words, we are interested in bifurcations where a healthy equilibrium loses stability and a toxic equilibrium becomes stable. For (3), a direct computation of the linearized system around the healthy equilibrium indicates that healthy and toxic equilibria interchange stability through a transcritical bifurcation occurring at $\kappa = 0$. Indeed, the stability of the healthy state is governed by a single eigenvalue

5 $$\begin{aligned} \lambda ^\textrm{H} = \frac{\kappa }{k_1} \end{aligned}$$

of the system’s Jacobian matrix evaluated at the healthy fixed point. Hence, we conclude that the healthy state is stable for $\kappa \le 0$ and the toxic fixed point is stable for $\kappa \ge 0$.

### The skewed heterodimer model

To understand the effect of activity dynamics on spreading, we now consider a constant activity $A\ge 0$ that affects the spreading as shown in Fig. 1b but exclude the effect that spreading may have on activity dynamics. Assuming that the activity process $A>0$ taking place in node 1 increases spreading to its neighbor, we obtain a *skewed heterodimer model* where the concentrations evolve according to

6a $$\begin{aligned} \dot{u}_1&= - (\ell + A) u_1 + \ell u_2 + k_0 - k_1 u_1 - k_2 u_1 v_1, \end{aligned}$$

6b $$\begin{aligned} \dot{v}_1&= -(\ell + A) v_1 + \ell v_2 - k_3 v_1 + k_2 u_1 v_1, \end{aligned}$$

6c $$\begin{aligned} \dot{u}_2&= (\ell + A) u_1 - \ell u_2 + k_0 - k_1 u_2 - k_2 u_2 v_2, \end{aligned}$$

6d $$\begin{aligned} \dot{v}_2&= (\ell + A) v_1 - \ell v_2 - k_3 v_2 + k_2 u_2 v_2. \end{aligned}$$

If $A=0$ we recover (3). For the skewed heterodimer model (6), there is a single healthy fixed point

7a $$\begin{aligned}&u_1^\textrm{H} = \frac{k_0 ( 2\ell + k_1 )}{k_1 (2\ell + A + k_1)}, \quad u_2^\textrm{H} = \frac{k_0 ( 2 (\ell +A) + k_1 )}{k_1 (2\ell + A + k_1)}, \quad v_1^\textrm{H} =v_2^\textrm{H}= 0. \end{aligned}$$

Note that introducing *A* breaks the symmetry in the healthy fixed point between the two nodes, which previously were independent of $\ell$. Eliminating $u_1,u_2$ and $v_1$ from the first fixed points, we find a cubic equation for the toxic fixed point ($v_2^\textrm{T}\not =0$) given by

$$\begin{aligned} c_0+c_1 v_2+c_2 v_2^2+c_3 v_2^3=0 \end{aligned}$$

with coefficient values given in “Appendix A”.

**Fig. 2:**
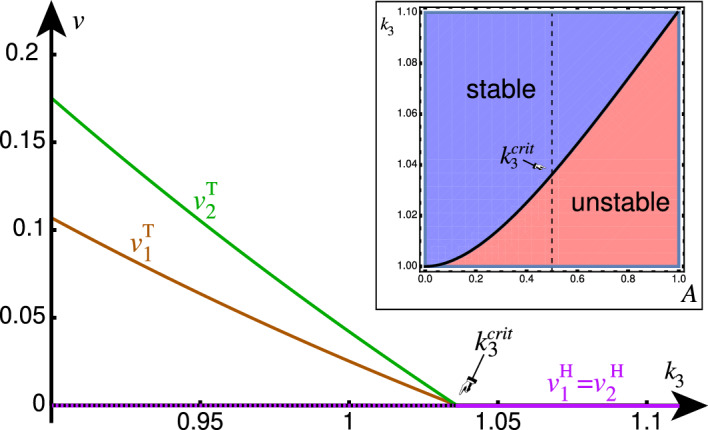
Bifurcation diagram for toxic load in nodes 1 and 2 as a function of toxic clearance $k_3$; other parameters are $A=1/2, \ell =1, k_0=1, k_1=1, k_2=1$. Inset: Bifurcation in (*A*,$k_3$) parameter space. Increasing activity destabilizes the healthy fixed point by shifting the transcritical bifurcation

To identify transitions between healthy and toxic states, we linearize the vector field at the healthy fixed point. The eigenvalues of the Jacobian at the healthy fixed point are

8a $$\begin{aligned} \lambda _1&= -k_1-2\ell -A,&\lambda _2&= - k_1, \end{aligned}$$

8b $$\begin{aligned} \lambda _3&= \frac{\kappa -\zeta }{k_1},&\lambda _4&= \frac{\kappa +\kappa ^{\text {crit}}}{k_1}, \end{aligned}$$

where $\kappa ^{\text {crit}}$ and $\zeta$ are given by

9a $$\begin{aligned} \kappa ^{\text {crit}}&= k_1 (2\ell +A)\frac{\sqrt{s_0^2 + s_1}-s_0}{2 s_0}, \end{aligned}$$

9b $$\begin{aligned} \zeta&= k_1 (2\ell +A)\frac{\sqrt{s_0^2 + s_1}+s_0}{2 s_0}, \end{aligned}$$

with constants $s_0 = k_1 (2\ell +A)(2\ell +A+k_1)$, $s_1 = 4 A^2 k_0 k_2 (k_1(2\ell +A+k_1) + k_0 k_2)$. Since all parameters are positive, it follows that $\kappa ^{\text {crit}} \ge 0$ and $\zeta \ge 0$. As such, we have that $\lambda _3 \le \lambda _4$ and $\lambda _1< \lambda _2 < 0$, and hence $\lambda _4$ dictates the stability of the healthy fixed point. The fixed point switches stability at a critical value $\kappa =-\kappa ^{\text {crit}}$, from which we can easily separate $k_3$. Freezing all parameters but the toxic clearance rate $k_3$, we look at the bifurcation in terms of the parameter $k_3$, with critical value

10 $$\begin{aligned} k_3^{\text {crit}} = \frac{k_0 k_2+\kappa ^{\text {crit}}}{k_1}, \end{aligned}$$

which satisfies ${k_0 k_2}/{k_1} \le k_3^{\text {crit}} \le 2 {k_0 k_2}/{k_1}$ and is monotonically increasing in *A* (see “Appendix B”). We conclude that introducing the activity parameter *A* shifts the transcritical bifurcation to higher values with respect to $k_3$. The effect of activity is to *destabilize* the healthy fixed point as shown in Fig. 2. Equivalently, in terms of neuroscientific applications, heterogeneous neuronal activity pushes neurons toward pathology.

It is interesting to understand the behavior of the toxic equilibrium as a function of the activity. Assuming that activity *A* is small compared to $\ell$, we can expand the toxic equilibrium to first order in *A* to obtain

11a $$\begin{aligned} u_1^\textrm{T}&= \frac{k_3}{k_2}\left( 1 + \frac{ \kappa -2 k_3 \ell }{ 4 k_3\ell ^2+2 k_0 k_2\ell + k_3 \kappa }A \right) + \mathcal {O}(A^2), \end{aligned}$$

11b $$\begin{aligned} u_2^\textrm{T}&= \frac{k_3}{k_2}\left( 1 - \frac{ \kappa -2 k_3 \ell }{ 4 k_3\ell ^2+2 k_0 k_2\ell + k_3 \kappa }A \right) + \mathcal {O}(A^2), \end{aligned}$$

11c $$\begin{aligned} v_1^\textrm{T}&= \frac{\kappa }{k_2 k_3} \left( 1 - \frac{2 k_3 \ell +k_3^2+k_0 k_2}{4 k_3\ell ^2+2 k_0 k_2\ell + k_3 \kappa }A \right) + \mathcal {O}(A^2), \end{aligned}$$

11d $$\begin{aligned} v_2^\textrm{T}&= \frac{\kappa }{k_2 k_3} \left( 1 + \frac{2 k_3 \ell +k_3^2+k_0 k_2}{4 k_3\ell ^2+2 k_0 k_2\ell + k_3 \kappa }A \right) + \mathcal {O}(A^2), \end{aligned}$$

**Fig. 3:**
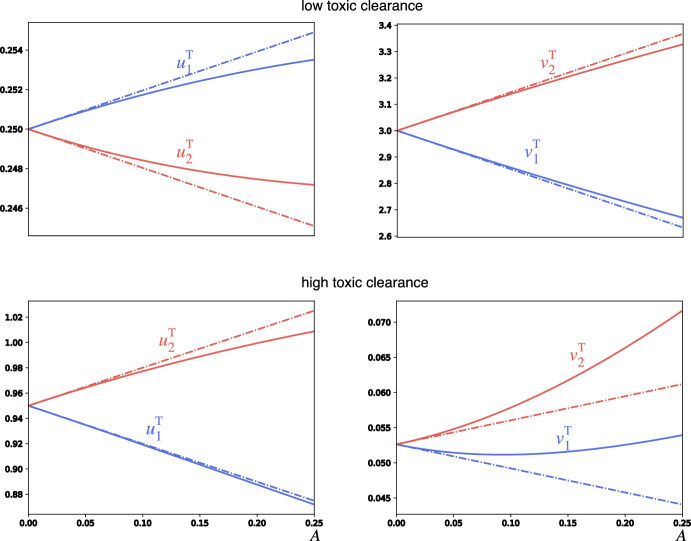
Comparison of steady-states of healthy and toxic species in nodes 1 (black) and 2 (red) determined by simulation (solid lines) and first-order Taylor expansion (stippled line) of the activity parameter *A*. The upper row demonstrates the conversion-dominated regime, whereas the bottom row demonstrates the shunting-dominated regime. All parameters are set to 1, except for $k_3=0.25$ in the first row (far from the transcritical bifurcation at $k_3^{\text {crit}} = 1$) and $k_3=0.95$ in the second row (close to the transcritical bifurcation) (color figure online)

We see that activity can affect the fixed point in two distinct ways assuming $\kappa >0$ so that the healthy fixed point is unstable: If $\kappa - 2 k_3 \ell > 0$, then $u_1$ increases while $v_1$ decreases and $u_2$ decreases while $v_2$ increases. By contrast, if $\kappa - 2 k_3 \ell < 0$ is small, then $u_1$ decreases while $v_1$ decreases, and $u_2$ increases while $v_2$ increases. In the former case, the conversion process is dominating, and the effective conversion at node 1 has decreased while it has increased in node 2. In the latter case, the transport process dominates and both species at node 1 are being shunted over to node 2. This *shunting* phenomenon does not occur in the original heterodimer model and is showcased in Fig. 3.

## Coupling heterodimer dynamics with oscillatory activity

In the previous analysis, we considered the activity *A* to be a constant. In the brain, activity may relate to collective neural oscillations that are fast compared to the disease progression. Therefore, we now assume that *A* is determined by the evolution of a pair of phase oscillators with Kuramoto coupling. Since the spreading and activity processes evolve on different time scales, the coupling between the two systems defines a slow-fast dynamical system.

### Two coupled phase oscillators

First, consider two phase oscillators, one on each node, with Kuramoto coupling. That is, the state of the oscillation on node $i\in \{1,2\}$ is given by a phase $\theta _i\in \mathbb {S}:= \mathbb {R} / 2\pi \mathbb {Z}$ that evolves according to

12a $$\begin{aligned} \dot{\theta }_1&= \omega _1 + \frac{K}{2} \sin {(\theta _2 - \theta _1)}, \end{aligned}$$

12b $$\begin{aligned} \dot{\theta }_2&= \omega _2 + \frac{K}{2} \sin {(\theta _1 - \theta _2)}, \end{aligned}$$

where $\omega _{i} > 0$ are the intrinsic frequencies of the nodes and $K \ge 0$ is the *coupling strength*. Since the coupling depends solely on the phase difference, the dynamics are completely determined by the evolution of the phase difference $\phi := \theta _1 - \theta _2$ determined by

13 $$\begin{aligned} \dot{\phi } = \Delta \omega - K \sin {\phi }, \end{aligned}$$

where $\Delta \omega = \omega _1 - \omega _2$ is assumed to be positive, without loss of generality. For $K > |\Delta \omega |$ there are two fixed points (one unstable and one stable attracting all initial conditions except the unstable fixed point). For $K < |\Delta \omega |$ there are no fixed points and any solution $\phi (t)$ is periodic. At the critical coupling strength $K=|\Delta \omega |$, there is a saddle-node bifurcation. Hence, we essentially have two regimes depending on the dynamics; we refer to them as the strong-coupling regime (fixed points) and the weak-coupling regime (periodic orbit).

We assume that the activity at each node is related to the *instantaneous frequencies*, $\dot{\theta }_1$ and $\dot{\theta }_2$ of each node. We define the *average frequency* of each oscillator:

14 $$\begin{aligned} \Omega _i = \lim _{T \rightarrow \infty } \frac{1}{T} \int _0^T \dot{\theta }_i(t) \text {d}t, \end{aligned}$$

which is independent of the initial conditions. In the strong-coupling regime (the coupling between oscillators is strong compared to the frequency mismatch and the phase difference $\phi$ converges to a fixed point), the oscillators are frequency locked. At the fixed points, we have a constant instantaneous frequency ${\dot{\theta }}_1(t) ={\dot{\theta }}_2(t)= \langle \omega \rangle :=(\omega _1 + \omega _2)/2$, which implies

15 $$\begin{aligned} \Omega _1 = \Omega _2= \langle \omega \rangle . \end{aligned}$$

In the weak-coupling regime (the coupling between the oscillators is weak compared to their frequency mismatch and the phase difference undergoes periodic oscillations), we compute the average frequencies $\Omega _i$ through the average frequency difference

16 $$\begin{aligned} \Delta \Omega = \lim _{T \rightarrow \infty } \frac{1}{T} \int _0^T \dot{\phi }(t) \text {d}t. \end{aligned}$$

Define $\Delta T = 2 \pi / \Delta \Omega$, assume $0 \le K < \Delta \omega$, and let $\phi (t)$ be the $\Delta T$-periodic solution of (13). Note that the sign of $\dot{\phi }$ is constant. With (13) we have

17 $$\begin{aligned} \Delta \Omega = \Delta \omega - K \lim _{T \rightarrow \infty } \frac{1}{T} \int _0^T \sin {\phi (t)} \text {d}t, \end{aligned}$$

With $m = T / |\Delta T|$, we can rewrite the integral as

18 $$\begin{aligned} \lim _{m \rightarrow \infty } \frac{1}{m |\Delta T|} \int _0^{m |\Delta T|} \sin {\phi (t)} \text {d}t= &  \frac{1}{|\Delta T|} \int _0^{|\Delta T|} \sin {\phi (t)} \text {d}t \nonumber \\= &  \frac{\Delta \Omega }{K} \left( \frac{\Delta \omega }{\sqrt{\Delta \omega ^2 - K^2}} - 1 \right) , \end{aligned}$$

where the last equality follows from substituting *t* by $\phi$ (which is possible since the sign of $\dot{\phi }$ is constant) and solving the resulting integral by Weierstrass substitution. Using this last expression in (17) yields

19 $$\begin{aligned} \Delta \Omega = \sqrt{\Delta \omega ^2 - K^2}, \end{aligned}$$

from which we compute the asymptotic frequencies of each node

20a $$\begin{aligned} \Omega _1&= \langle \omega \rangle + \frac{\sqrt{\Delta \omega ^2 - K^2 }}{2}, \end{aligned}$$

20b $$\begin{aligned} \Omega _2&= \langle \omega \rangle - \frac{\sqrt{\Delta \omega ^2 - K^2 }}{2}. \end{aligned}$$

### Slow-fast heterodimer-oscillator dynamics

Next, we couple the oscillatory dynamics with the heterodimer model of protein spreading. The two processes will evolve on distinct time scales, determined by a small strictly positive constant $\epsilon \ll 1$, representing the ratio between the fast activity time scale and the slow spreading time scale. Specifically, the two-node *heterodimer-oscillator* (see Fig. 1c) system is

21a $$\begin{aligned} \dot{u}_1&= - (\ell + \delta A_1(\phi )) u_1 + (\ell + \delta A_2 (\phi )) u_2 + k_0 - k_1 u_1 - k_2 u_1 v_1, \end{aligned}$$

21b $$\begin{aligned} \dot{v}_1&= -(\ell + \delta A_1(\phi )) v_1 + (\ell + \delta A_2(\phi )) v_2 - k_3 v_1 + k_2 u_1 v_1, \end{aligned}$$

21c $$\begin{aligned} \dot{u}_2&= (\ell + \delta A_1(\phi )) u_1 - (\ell + \delta A_2(\phi )) u_2 + k_0 - k_1 u_2 - k_2 u_2 v_2, \end{aligned}$$

21d $$\begin{aligned} \dot{v}_2&= (\ell + \delta A_1(\phi )) v_1 - (\ell + \delta A_2(\phi )) v_2 - k_3 v_2 + k_2 u_2 v_2, \end{aligned}$$

21e $$\begin{aligned} \epsilon \dot{\theta }_1&= \widehat{\omega }_1(v_1) + \frac{K}{2} \sin {(\theta _2 - \theta _1)}, \end{aligned}$$

21f $$\begin{aligned} \epsilon \dot{\theta }_2&= \widehat{\omega }_2(v_2) + \frac{K}{2} \sin {(\theta _1 - \theta _2)}, \end{aligned}$$

where $\delta > 0$ scales the oscillators’ effect on spreading. We assume that the coupling between heterodimer and oscillatory dynamics is through the phase-dependent activity of nodes 1 and 2, that is,

22 $$\begin{aligned} A_1(\phi ) = \epsilon \dot{\theta }_1,\quad A_2(\phi ) = \epsilon \dot{\theta }_2, \end{aligned}$$

and the intrinsic frequencies

23 $$\begin{aligned} \widehat{\omega }_1(v_1) = \omega _1 - c v_1,\quad \widehat{\omega }_2(v_2) = \omega _2 - c v_2, \end{aligned}$$

that are decreased by the presence of toxic proteins with a scaling parameter $c > 0$. As discussed above, we may replace the phase dynamics in (21) by the evolution of the phase difference $\phi$ as above given by

24 $$\begin{aligned} \epsilon \dot{\phi }&= \Delta \omega - c \Delta v - K \sin {\phi }, \end{aligned}$$

where $\Delta v = v_1 - v_2$ is the difference in toxic protein concentration. The phase locking behavior is now determined by the effective intrinsic frequency difference $\Delta \widehat{\omega } = \widehat{\omega }_1 - \widehat{\omega }_2 = \Delta \omega - c \Delta v$, which is a function of $\Delta v$. As there is no sensible interpretation of *negative* neuronal activity, we will only consider parameters for which $A_i(t) \ge 0, i \in \{1,2\}$ for all *t*, which implies that the intrinsic frequencies are positive $\omega _i>0$.

Given that the spreading dynamics is much slower than the oscillator dynamics (on the order of years versus seconds), we are interested in the dynamics for small $\epsilon$ close to the singular limit $\epsilon \rightarrow 0$. In the singular limit, the phase dynamics relax instantaneously to the asymptotic dynamics of the phase-difference $\phi (t)$. Thus, the dynamics in the singular limit depend on which dynamical regime the phase difference is operating in. In the phase-locked regime, the dynamics relax instantaneously to equilibrium, which defines the critical manifold of the slow-fast system on which $A_{i}$ takes its value at equilibrium. In the regime where the phase difference $\phi (t)$ is drifting, we replace the instantaneous frequency in $A_{i}$ by the temporal average $\Omega _i$; this is similar to the approach in Thiele et al. (2023). Finally, we consider the system at the border between the two regimes.

### The phase-locking regime

Assume that $|\Delta \widehat{\omega }(\Delta v)| \le K$. Then the singular-limit dynamics on the slow manifold is determined by the stable phase-difference equilibria

25 $$\begin{aligned} \phi = \sin ^{-1}{\left( \frac{\Delta \omega - c\Delta v}{K} \right) }. \end{aligned}$$

Inserting the fixed point into $A_i$, both nodes have identical activities

26 $$\begin{aligned} A_1 = A_2 = \langle \omega \rangle - \frac{c(v_1+v_2)}{2} . \end{aligned}$$

Substituting these expressions into the slow system gives us the dynamics on the phase-locking critical manifold.

Since the nodes have identical activity levels, the dynamics are qualitatively equivalent to those of the isolated heterodimer model. In particular, the system has the same pair of healthy and toxic fixed points as the heterodimer model given by (4):

27a $$\begin{aligned}&u_1^{\textrm{H},\textrm{P}} = u_2^{\textrm{H},\textrm{P}} = \frac{k_0}{k_1},\quad v_1^{\textrm{H},\textrm{P}} = v_2^{\textrm{H},\textrm{P}} = 0, \end{aligned}$$

27b $$\begin{aligned}&u_1^{\textrm{T},\textrm{P}} = u_2^{\textrm{T},\textrm{P}} = \frac{k_3}{k_2},\quad v_1^{\textrm{T},\textrm{P}} = v_2^{\textrm{T},\textrm{P}} = \frac{\kappa }{k_2 k_3}. \end{aligned}$$

The stability of the healthy fixed point is determined by the eigenvalues of the Jacobian matrix:

28a $$\begin{aligned} \lambda _1&= - k_1 - 2 (\ell + \delta \langle \omega \rangle ),\quad \lambda _2 = - k_1, \end{aligned}$$

28b $$\begin{aligned} \lambda _3&= \frac{\kappa }{k_1} - 2(\ell + \delta \langle \omega \rangle ), \quad \lambda _4 = \frac{\kappa }{k_1}. \end{aligned}$$

Thus, the healthy fixed point loses its stability at $\kappa =0$.

The assumption of being in the phase-locking regime, $|\Delta \widehat{\omega }(\Delta v)| < K$, gives a consistency condition for the existence of the fixed points on the critical manifold. Note that since the activity of each node in the phase-locking regime is identical, the slow dynamics is symmetric in the sense that exchanging the two nodes has no impact on the dynamics. Furthermore, for both fixed points, the nodes are “equal” in the sense that they take the same state and satisfy $\Delta v = 0$. Thus, the healthy and toxic fixed points only exist as fixed points on the critical manifold for the slow dynamics if $|\Delta \widehat{\omega }(0)| = |\Delta \omega | \le K$.

### The drifting regime

Outside the phase-locked regime, $|\Delta \widehat{\omega }(\Delta v)| > K$, the fast oscillatory dynamics do not relax to equilibrium but evolve on a periodic orbit. As these oscillations are much faster than the evolution of the slow dynamics, we average out the fast oscillations by replacing the activities $A_i$ by their temporal averages to define the *drifting regime*. Specifically, replacing $\omega _i$ with $\widehat{\omega }_i(v_i)$ in (20) and assuming, without loss of generality, that $\Delta \widehat{\omega } \ge 0$ yields the activities

29a $$\begin{aligned} A_1(v) := \epsilon \widehat{\Omega }_1&= \langle \omega \rangle - \frac{ c (v_1 + v_2)- \sqrt{(\Delta \widehat{\omega }(\Delta v))^2 - K^2}}{2}, \end{aligned}$$

29b $$\begin{aligned} A_2(v) := \epsilon \widehat{\Omega }_2&= \langle \omega \rangle - \frac{ c (v_1 + v_2) + \sqrt{(\Delta \widehat{\omega }(\Delta v))^2 - K^2}}{2}. \end{aligned}$$

Substituting these activities into the dynamical equations for the slowly-evolving heterodimer equations yields the dynamics of the drifting regime. As the activities of the two nodes are now distinct, the dynamics is similar to the skewed heterodimer model in Sect. 2.2. There is one healthy fixed point $u^{\textrm{H},\textrm{D}}$ in the drifting regime with coefficients

30a $$\begin{aligned} u_1^{\textrm{H},\textrm{D}}&= \frac{k_0}{k_1} \left( 1 - \delta \frac{ \sqrt{\Delta \omega ^2-K^2}}{k_1 + 2\ell + 2\delta \langle \omega \rangle } \right) , \end{aligned}$$

30b $$\begin{aligned} u_2^{\textrm{H},\textrm{D}}&= \frac{k_0}{k_1} \left( 1 + \delta \frac{ \sqrt{\Delta \omega ^2-K^2}}{k_1 + 2\ell + 2\delta \langle \omega \rangle } \right) , \end{aligned}$$

30c $$\begin{aligned} v_1^{\textrm{H},\textrm{D}}&= v_2^{\textrm{H},\textrm{D}} = 0, \end{aligned}$$

under the assumption that $|\Delta \widehat{\omega }(0)|=|\Delta \omega | \ge K$. Similar to the skewed heterodimer model, the symmetry of the fixed points is broken. Linear stability of the healthy fixed point is determined by the eigenvalues of the Jacobian matrix:

31a $$\begin{aligned} \lambda _1&= -k_1 - 2(\ell + \delta \langle \omega \rangle ), \quad \lambda _2 = -k_1, \end{aligned}$$

31b $$\begin{aligned} \lambda _3&= \frac{\kappa - \zeta }{k_1}, \quad \lambda _4 = \frac{\kappa + \kappa ^{\text {crit}} }{k_1}, \end{aligned}$$

where

$$\begin{aligned} \kappa ^{\text {crit}}&= k_1 (\ell +\delta \langle \omega \rangle ) \frac{\sqrt{s_0^2 + s_1}-s_0}{s_0}, \\ \zeta&= k_1 (\ell +\delta \langle \omega \rangle ) \frac{\sqrt{s_0^2 + s_1}+s_0}{s_0}, \end{aligned}$$

with $s_0 = 2 k_1 \left( \ell + \delta \langle \omega \rangle \right) (k_1+2\ell +2 \delta \langle \omega \rangle )$ and $s_1 = 4 \delta ^2 k_0 k_2 (\Delta \omega ^2-K^2) (k_0 k_2 +k_1\left( k_1+2\ell +2\delta \langle \omega \rangle \right) )$. Remembering that the healthy fixed point only exists for $|\Delta \omega | > K$, we can assert that $\kappa ^{\text {crit}}, \zeta > 0$, as is verifiable by inspecting $s_1$. As such, $\lambda _4$ determines the stability of $u^{\textrm{H},\textrm{D}}$. The critical value for $k_3^{\text {crit}}$ at which the transcritical bifurcation occurs is

32 $$\begin{aligned} k_3^{\text {crit}} = \frac{k_0 k_2 + \kappa ^{\text {crit}}}{k_1}. \end{aligned}$$

Assuming $\delta$ small compared to $\ell$, we expand the toxic fixed point $u^{\textrm{T},\textrm{D}}$ in the drifting regime to first order in $\delta$, giving us

33a $$\begin{aligned} u_1^{\textrm{T},\textrm{D}}&= \frac{k_3}{k_2} \left( 1 + \delta \frac{ \left( \kappa -2 k_3 \ell \right) \sqrt{\Delta \omega ^2 - K^2}}{4 k_3\ell ^2+2 k_0 k_2\ell + k_3 \kappa } \right) + \mathcal {O}(\delta ^2), \end{aligned}$$

33b $$\begin{aligned} u_2^{\textrm{T},\textrm{D}}&= \frac{k_3}{k_2} \left( 1 - \delta \frac{ \left( \kappa -2 k_3 \ell \right) \sqrt{\Delta \omega ^2 - K^2}}{4 k_3\ell ^2+2 k_0 k_2\ell + k_3 \kappa } \right) + \mathcal {O}(\delta ^2), \end{aligned}$$

33c $$\begin{aligned} v_1^{\textrm{T},\textrm{D}}&= \frac{\kappa }{k_2 k_3} \left( 1 - \delta \frac{ \left( 2 k_3 \ell +k_3^2+k_0 k_2\right) \sqrt{\Delta \omega ^2 - K^2}}{4 k_3\ell ^2+2 k_0 k_2\ell + k_3 \kappa } \right) + \mathcal {O}(\delta ^2), \end{aligned}$$

33d $$\begin{aligned} v_2^{\textrm{T},\textrm{D}}&= \frac{\kappa }{k_2 k_3} \left( 1 + \delta \frac{ \left( 2 k_3 \ell +k_3^2+k_0 k_2\right) \sqrt{\Delta \omega ^2 - K^2}}{4 k_3\ell ^2+2 k_0 k_2\ell + k_3 \kappa } \right) + \mathcal {O}(\delta ^2), \end{aligned}$$

where the coefficients are similar to the expansion of the skewed heterodimer toxic fixed point, except that they are scaled by $\sqrt{\Delta \omega ^2 - K^2}$. As such, we have transport- and conversion-dominated behavior for small and large values of $\kappa - 2 k_3 \ell$ respectively. More importantly, we have established the existence of a toxic fixed point $u^{\textrm{T},\textrm{D}}$ on the drifting regime for small $\delta$.

**Fig. 4:**
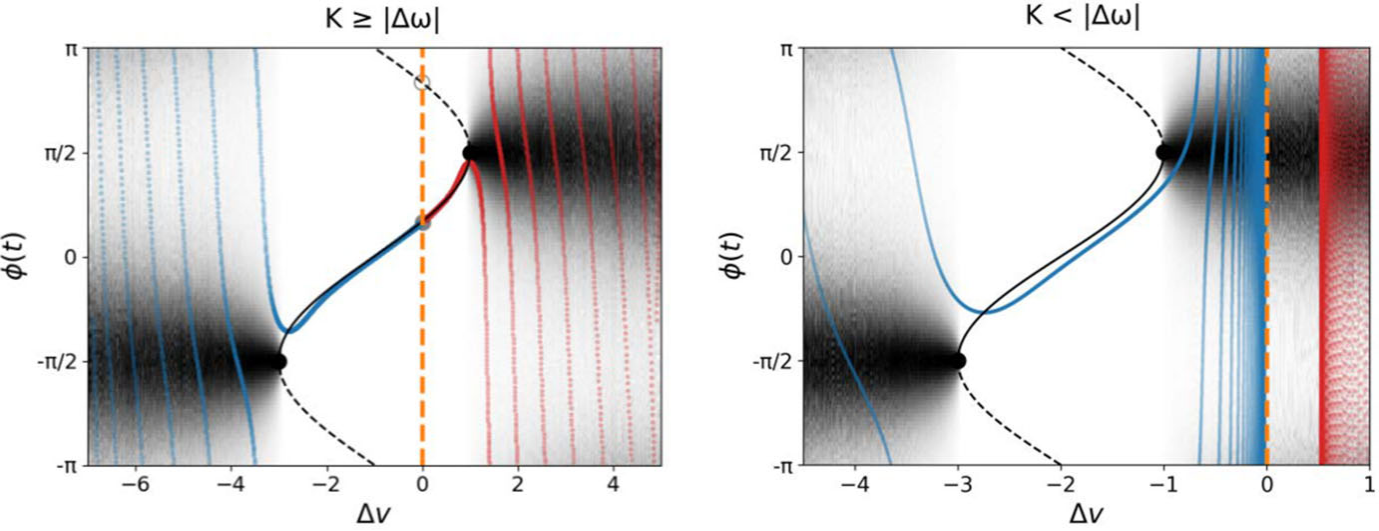
Summary of the dynamics in the phase-locking and drifting regime with simulations in the healthy (black) and toxic (red) regimes of the heterodimer-oscillator with $\epsilon > 0$. *Left:* Summary for $K > \Delta \omega$ over the phase-difference and toxic species difference, where $K=2, \Delta \omega =1, c=1, k_0=1, k_1= 1, k_2= 1, \delta =1, \ell = 10^{-3}$ with forward solutions in the toxic ($\epsilon =0.2$, $k_3=0.75$) and healthy regime ($\epsilon =0.075$, $k_3=1.25$). Both forward solutions are symmetric with respect to the slow variables. *Right:* Summary for $K < \Delta \omega$ where $K=1, \Delta \omega =2, c=1, k_0=1.5, k_1= 1, k_2= 1, \delta =1, \ell = 10^{-3}$ and with forward solutions in toxic ($\epsilon =0.1, k_3=0.125$) and healthy regimes ($\epsilon =0.075,k_3=1.25$). Both forward solutions are asymmetric with respect to the slow variables (the healthy solution is asymmetric with respect to the healthy species). Note that the trajectories in the healthy regimes converge to $\Delta v = 0$ (highlighted with a stippled, orange line) in both diagrams (color figure online)

**Fig. 5:**
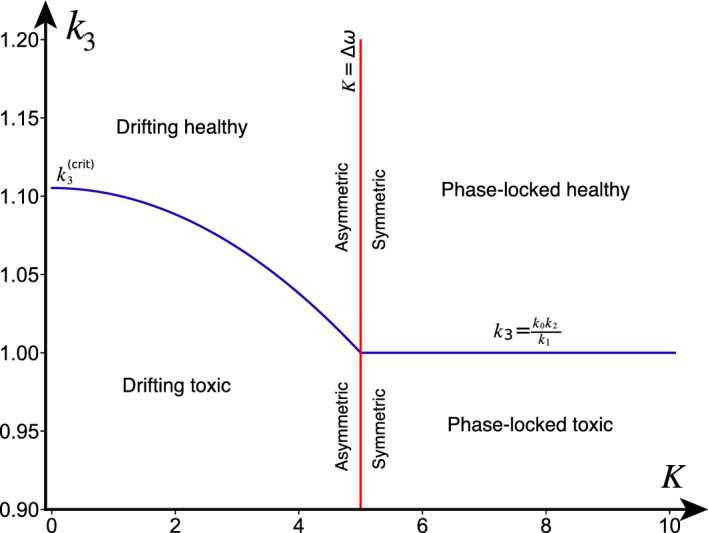
Summary of the dynamics of the 2-node heterodimer-oscillator in the singular limit ($\epsilon \rightarrow 0$). The labels in each quadrant state which fixed point we know to be stable. The transcritical bifurcation in the weak-coupling and strong-coupling regime is presented, together with the breaking of the symmetry between the two nodes in the fixed points, which occurs at $K=|\Delta \omega |$. Parameters are $k_0=1, k_1=1, k_2=1, \ell = 1, \omega _1=10, \omega _2=5, \delta =5$

Note that the above coefficients are only defined for $K \le |\Delta \omega |$, similarly to $u^{\textrm{H},\textrm{D}}$. As such, our preceding analysis suggests a symmetry-breaking, global bifurcation occurring at $K = |\Delta \omega |$ in which from one side (from the phase-locking regime) two fixed point branches collide and disappear (saddle-node bifurcation on an invariant circle), but from the other side (from the drifting regime) two periodic solutions collide and disappear. Furthermore, the fixed points in the phase-locking regime are symmetric between the nodes with respect to their heterodimer variables, whereas both the periodic solutions are asymmetric in this respect. A summary of the heterodimer-oscillator dynamics in the strong-coupling and weak-coupling regimes can be found in Fig. 4 alongside numerical solutions for $\epsilon > 0$. Moreover, an overview of the dynamical regimes and the (singular-limit) transcritical bifurcation is illustrated in $(K,k_3)$ parameter space in Fig. 5.

### Transitions between the phase-locking and drifting regimes

With an understanding of the dynamics within the phase-locking regime (Sect. 3.3) and drifting regime (Sect. 3.4) at hand, we can now elucidate possible transitions between the regimes. The boundary between the regimes is where the fast dynamics undergo a saddle-node bifurcation at $|\Delta \widehat{\omega }(\Delta v)| = |\Delta \omega - c \Delta v| = K$. Equivalently, we obtain the following condition for the regime border

34 $$\begin{aligned} \Delta v = \frac{\Delta \omega \pm K}{c}. \end{aligned}$$

**Fig. 6:**
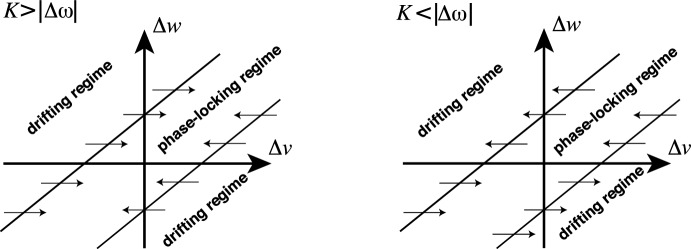
The vector field of $\Delta v$ in terms of $\Delta \omega$ and $\Delta v$ in the strong-coupling (left) and weak-coupling regime (right). The inner region in both diagrams is the phase-locking regime, and the outer regions are the drifting regime. We see that for strong coupling $K > |\Delta \omega |$, the vector field points inwards to the phase-locking regime. However, for the weak-coupling regime $K < |\Delta \omega |$, the vector field points to the left-hand drifting regime for $\Delta \omega > 0$ (node 1 is more active than node 2) and to the right-hand drifting regime for $\Delta \omega < 0$ (node 2 is more active than node 1)

The value of $\Delta v$ is subject to the slow dynamics (21). Specifically, the sign of  determines the transitions between the phase-locking and drifting regimes: For the right boundary of the phase-locking regime, $\Delta v = \frac{\Delta \omega + K}{c}$, negative  indicates that the slow flow points from the drifting regime into the phase-locking regime and a positive  in the opposite direction. For the left boundary, the conditions are the other way around. In the following, we will argue that, under certain assumptions, the flow points towards the phase-locking regime for $K > |\Delta \omega |$ and towards the drifting regime for $K < |\Delta \omega |$; this is sketched in Fig. 6.

To determine the transitions between the regimes, we consider the dynamics of $\Delta v$. In the singular limit, the dynamics have relaxed to the saddle-node equilibrium and thus $A^*:= A_1 = A_2$. Now we assume that the $u_i$ take their equilibrium values, with $u_1 = u_2 = k_0 / k_1$ (healthy regime) and $u_1 = u_2 = k_3 / k_2$ (toxic regime); $u_1 = u_2 =: u^*$ in either case. According to (21), the evolution of $\Delta v$ is determined by35We claim that the first factor is not positive (i.e., the quantity in the parentheses is not negative). The first two terms are clearly positive since $\ell \ge 0$ and, by assumption, $A^* \ge 0$. For the third term, $k_3 - k_2u^*\ge 0$ is equivalent to $u^* \le \frac{k_3}{k_2}$. But, by assumption, $u^*=k_3 / k_2$ or $u^* = k_0 / k_1 \le k_3/k_2$ so in either case the third term is not negative. We conclude that the sign of  only depends on the sign of $\Delta v$. For the right boundary of the phase-locking regime, we have $\Delta v = (\Delta \omega + K)/c$ so $\Delta v > 0$ or equivalently $K>-\Delta \omega$ implies  (flow towards the phase-locking regime). Conversely, $K<-\Delta \omega$ implies  (flow towards the drifting regime). Similarly, for the left boundary of the phase-locking regime, we have $\Delta v = (\Delta \omega - K)/c$ so $K<\Delta \omega$ implies  (flow towards the drifting regime) and $K>\Delta \omega$ implies  (flow towards the phase-locking regime).

Thus in terms of the system parameters, the crucial quantity is the oscillator coupling relative to the intrinsic frequency mismatch. If $K>|\Delta \omega |$ then the flow points towards the phase-locking regime on either boundary. If $K<|\Delta \omega |$ then the flow points in the same direction on each boundary and the direction is determined by the sign of $\Delta v$. These cases are illustrated in Fig. 6.

### Extending the parameter regime

From the beginning, we have assumed *c* and $\delta$ to be positive. These assumptions, however, may not be fit for all applications of the heterodimer-oscillator model. For example, one might envision spreading processes that *increases* oscillatory activity locally ($c < 0$), and, in return, oscillatory processes that *decreases* spreading to its neighboring oscillators ($\delta < 0$). First, we consider the case $c < 0$. None of the singular-limit fixed points nor their stability depend on *c*, and the regime border analysis above can be repeated successfully for $c<0$ and $\delta > 0$ (noting that the left- and right-hand borders swap places). For $\delta < 0$, we may assume $\delta \langle \omega \rangle > -\ell$ to guarantee that our stability analysis of the phase-locking and drifting equilibria remains unaffected [see eigenvalues in Eqs. (28) and (31)]. The assumption is within reason; it is equivalent to stating that the link between the nodes $\ell +\delta A(\phi )$ does not change signs for $\Delta v = 0$. For the regime border analysis to hold, we require $\delta \ge - 2 \ell / A^*_\text {max}$ where $A^*_\text {max}$ is the maximum of the phase-locked activity over $v_1$ and $v_2$ [see Eq. (26)]. For $c>0$, we have that $A^*_\text {max}= \langle \omega \rangle$ giving $\delta \langle \omega \rangle \ge - 2 \ell$, which is already satisfied by $\delta \langle \omega \rangle > -\ell$. However, $A^*_\text {max}$ increases indefinitely in $v_1$ and $v_2$ for $c<0$. Hence, we need additional bounds on the variables $v_1$ and $v_2$ to ensure that the regime border analysis holds. Although the regime border analysis cannot be repeated for $c, \delta <0$ without further assumptions, we can conclude that the fixed point linear stability analysis generalizes to $c, \delta \in \mathbb {R}$,

## Activity-spreading feedback on networks

Investigating the dynamics of the heterodimer-oscillator system on more general networks, we find that the results from the 2-node heterodimer-oscillator system provide a strong intuition for the generalized network dynamics. Specifically, we consider a network of *N* nodes determined by the $N\times N$ (weighted) adjacency matrix ${\textbf{W}}$ with Laplacian $\textbf{L}$. Let $u, v \in \mathbb {R}^N$ denote the healthy and toxic species concentration at each node and $\theta \in \mathbb {S}^N$ the state of the oscillators on each node. Generalizing (21), the states evolve according to

36a $$\begin{aligned} \dot{u}_i&= - \sum _{j=1}^N L_{ij} (1+\delta A_j) u_j + k_0 - k_1 u_i - k_2 u_i v_i, \text { for } 1 \le i \le N \end{aligned}$$

36b $$\begin{aligned} \dot{v}_i&= -\sum _{j=1}^N L_{ij} (1+\delta A_j) v_j-k_3 v_i + k_2 u_i v_i, \text { for } 1 \le i \le N \end{aligned}$$

36c $$\begin{aligned} \epsilon \dot{\theta }_i&= \omega _i - c v_i + K \sum _{j=1}^{N} W_{ij} \sin {(\theta _j - \theta _i)}, \text { for } 1 \le i \le N \end{aligned}$$

where $$A = \epsilon [ \dot{\theta }_1, \dot{\theta }_2, \dotsc , \dot{\theta }_N ] $$.

### Numerical exploration of key example networks

**Fig. 7:**
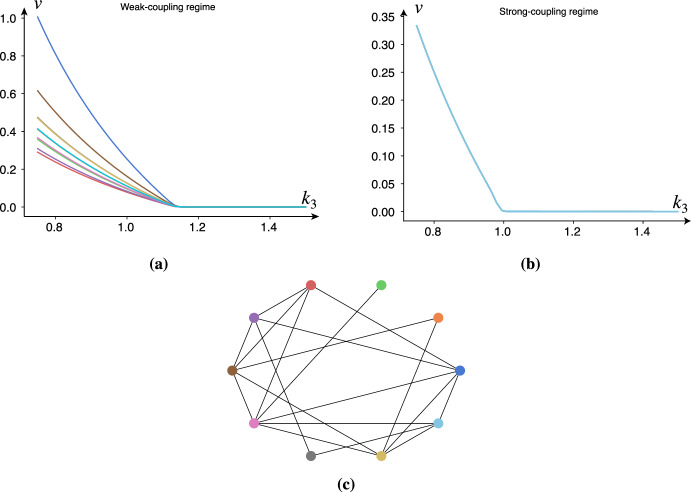
Simulations demonstrating the transcritical bifurcation during the weak-coupling and strong-coupling regime in a random graph. All weights in the network are set to 1, and the intrinsic frequencies were drawn from a normal distribution. **a** The weak-coupling parameters are $\rho =0.1, k_0=1, k_1=1, k_2=1, E(\omega )=10, \text {Var}(\omega )=0.25, c=0.5, \epsilon =10^{-3}, \delta =10, K=0.1$, while one of the oscillators (dark black) at a slower frequency $\omega =5$. **b** The strong-coupling parameters are $\rho =0.1, k_0=1, k_1=1, k_2=1, E(\omega )=10, \text {Var}(\omega )=0, c=0.5, \epsilon =10^{-3}, \delta =10, K=0.1$. **c** The Erdős–Rényi graph ($N=10, p=0.5$)

*Erdős–Rényi random graphs* Dynamics on Erdős–Rényi random graphs retain the transcritical bifurcation near $\kappa = 0$ alongside its symmetry for small differences between the intrinsic frequencies of the nodes; cf. Fig. 7. However, with large differences in the intrinsic frequencies, the transcritical bifurcation extends the toxic parameter regime and breaks the symmetry of the fixed points between the nodes, just as expected from our analysis of the 2-node system.

**Fig. 8:**
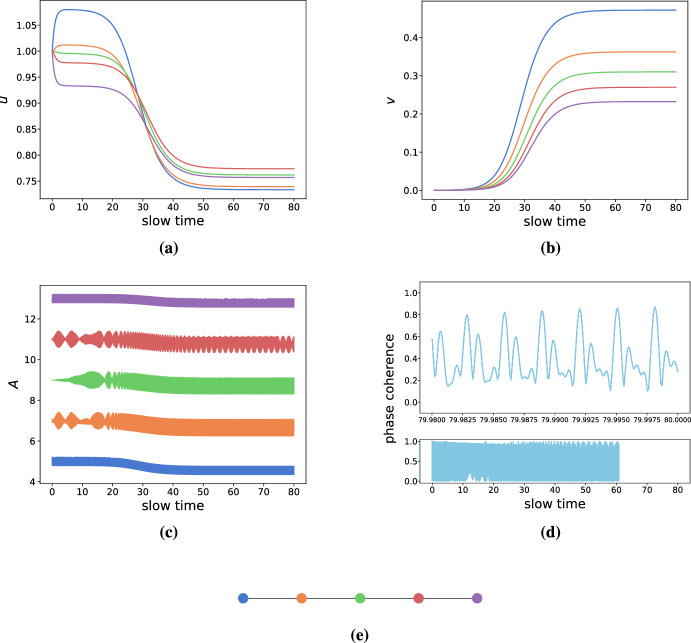
Simulations demonstrating heterodimer-oscillator dynamics on a chain network. All link weights are set to 0.1 while other parameters are $\rho =0.5, k_0=1, k_1=1, k_3=0.75, k_2=1, \epsilon =10^{-3}, \delta =1\, K=1, c=0.5$. The natural frequencies of the nodes, $\omega _i$, range from 5 to 15 with increments of 2.5. Colors are consistent across figure panels. **a** The evolution of healthy species. **b** The evolution of toxic species. **c** The activity (instantaneous frequencies) of the nodes. **d** The phase-coherence of the Kuramoto order parameter of the oscillators. **e** Graph of the network (color figure online)

*Chain graphs* To further test our intuition, we create a chain graph with decreasing frequencies along the chain. If we initialize a small amount of toxic species in each node, we expect (in the steady state) a gradient of increasing toxic species along the chain. As observed in Fig. 8, this prediction is accurate. Additionally, we observe shunting behavior. First, the healthy species are quickly transported according to the nodes’ activity gradient, and then the healthy species are converted into toxic species. According to our 2-node analysis, such shunting behavior should occur close to the original transcritical bifurcation $\kappa = 0$, which is where the simulation in Fig. 8 has been parameterized.

**Fig. 9:**
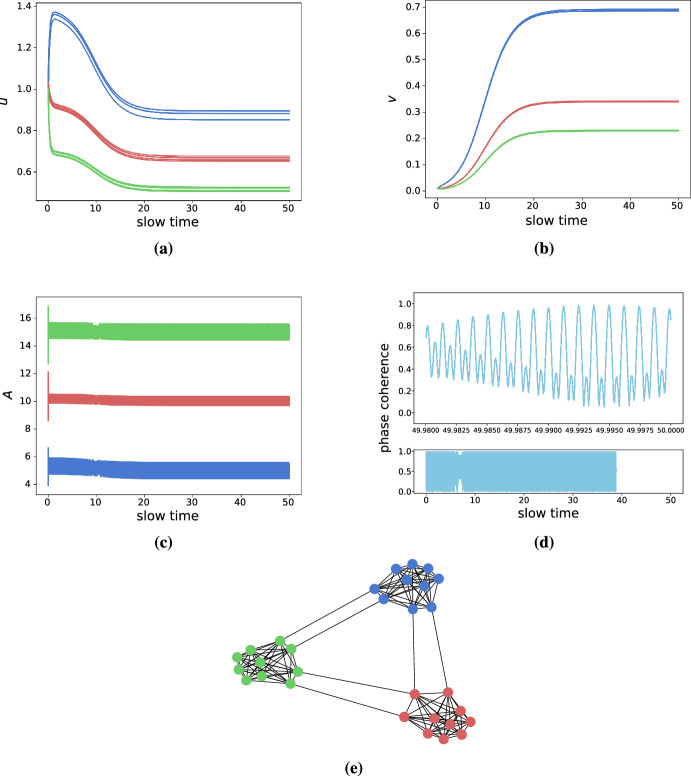
Simulations demonstrating heterodimer-oscillator dynamics on a clustered network. All network link weights are set to 0.1, while other parameters are $\rho =0.5, k_0=1, k_1=1, k_3=0.75, k_2=1, \epsilon =10^{-3}, \delta =1\, K=1, c=0.5$. Clusters are colored green (highest activity), red (medium activity), and red (lowest activity) respectively. The average intrinsic frequencies of the clusters, $\omega _i$, are normally distributed with means 5 (black), 10 (red), and 15 (green) and a common standard deviation of 0.5. **a** The evolution of healthy species. **b** The evolution of toxic species. **c** The activity (instantaneous frequencies) of the nodes **d** The phase-coherence of the Kuramoto order parameter of the oscillators. **e** Graph of the network (color figure online)

*Clustered networks* Many complex networks show high degrees of clustering. As such, we created a network of 3 fully-connected clusters of 10 nodes each, where each cluster is connected to each other by two links chosen between a random node pair; cf. Fig. 9. We then drew the intrinsic frequencies from normal distributions where each cluster has a different mean. That is, one cluster will be highly active, one will be moderately active, and one will be less active. By doing so, we will have 3 synchronized clusters that are weakly connected to each other. As before, we set the parameters in the toxic regime, yet close to the original transcritical bifurcation at $\kappa = 0$. As shown in Fig. 9, the simulations confirm the intuition from the 2-node system. At first, the healthy species are shunted towards the lesser active clusters, where they are subsequently converted into toxic species. The least active cluster thus produces the most toxic species followed by the moderately active and highly active clusters, respectively. These simulations suggest that the heterodimer-oscillator might also be suitable for mean-field models of population dynamics.

### Exploring coevolutionary dynamics in Alzheimer’s disease

Our original motivation was to investigate the effect that the slow-fast dynamics between neuronal activity and pathological protein spreading exert on the progression of neurodegenerative diseases. Previous studies have modeled the progression of Alzheimer’s disease as a spreading process on network reconstructions of the human brain. These network reconstructions are called connectomes and are built using DTI imaging which are subsequently parcellated into networks of arbitrary size. Nodes in the network represent brain regions and links between brain regions represent axonal bundles.

Typically, modelers will simulate the spreading of tau proteins across the connectome, leading to neurodegeneration and neuronal death. Tau proteins start to aggregate at the entorhinal cortex and spread progressively to the hippocampus, the limbic system, and the neocortex. The successive spread of tau has been shown to follow a pattern, and, as such, the spreading of tau is divided into six stages known as the Braak staging scheme. However, not all patients follow the Braak staging scheme. In fact, studies suggest that Alzheimer’s patients fit into different subgroups based on their staging patterns (Duits et al. 2021; Ferreira et al. 2020). Furthermore, as noted in the Introduction, tau proteins are believed to be transported at a higher rate from higher-active neurons (Wu et al. 2016), and several studies suggest a crucial link between brain-wide correlations of brain activity and disease spreading patterns (Seemiller et al. 2021; Franzmeier et al. 2020). We here provide proof-of-concept, with our heterodimer-oscillator model, that neuronal activity may play a mechanistic role in the spreading of tau protein seen in Alzheimer’s disease staging.

**Fig. 10:**
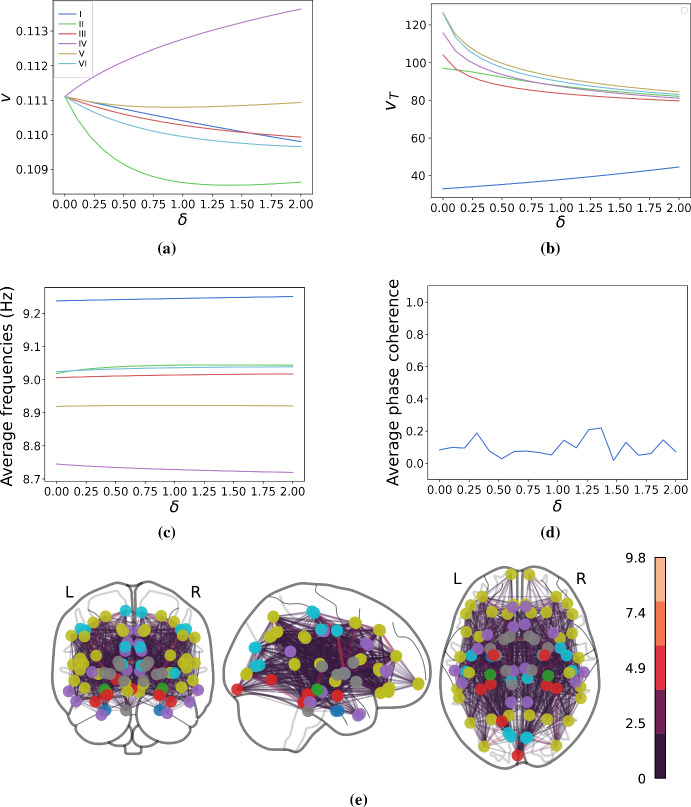
Simulations of toxic tau spreading across an 83-node human connectome showing the asymptotic (steady-state) behavior of the system as a function of $\delta$ (the effect activity has on spreading). Parameters are $\rho =0.001, k_0 =1, k_1=1, k_3=0.9, k_2=1, \epsilon =0.01, K=0.1, c=10$. The natural frequencies, $\omega _i$, are drawn from a normal distribution with a mean 10 and standard deviation of 0.5. Nodes are initialized with $u_i = 1$ and $v_i=0$, apart from the entorhinal cortices which are initialized with $u_i = 1$ and $v_i=0.1$ (nonzero toxic concentration). **a** Average asymptotic amount of tau species in each Braak stage. **b** Average time for regions in different Braak stages to become infected with tau. **c** Asymptotic average frequencies of the oscillators at the end of the slow-time simulation. **d** Average global phase-coherence over the entire slow-time spreading simulation. **e** Graph of the brain network with edges colored according to their weight. Nodes that are not part of any Braak stage are colored gray

We simulate the spreading of tau on the 83-node Budapest Reference Connectome (Szalkai et al. 2017)—in which the simulation initially follows the canonical Braak staging pattern—and gradually increase the effect that neuronal activity has on spreading, which is achieved by increasing $\delta$. To simulate the natural progression of Alzheimer’s disease, we only initialize a nonzero concentration of toxic protein in the entorhinal cortex (Braak stage I).

To visualize the Alzheimer’s simulations more easily, we investigate metrics averaged over the regions per their Braak staging. As shown in Fig. 10a, we see that neuronal activity induces symmetry breaking in asymptotic toxic protein concentrations (asymptotic refers here to the end of disease simulation). Different regions become more susceptible to tau pathology than others due to the activity-dependence of tau spreading. Furthermore, in Fig. 10b, we see that the arrival time of the Braak staging is affected by neuronal activity, although the ordering of the Braak stages is robust. Even without activity-dependent spreading, the tau spreading model achieves the correct Braak staging. Including neuronal activity swaps the order of Braak stage II and III, but the ordering remains unaffected otherwise.

However, the inclusion of activity-dependent spreading has little effect on the neuronal dynamics themselves, as evidenced in Fig. 10c, d. In Fig. 10c, we show the asymptotic average frequency of the neural oscillators. As tau spreads, it decreases the intrinsic frequencies of the oscillators. However, the amount of frequency slowing caused by the tau spreading is not impacted by its activity dependence. As such, there is little change in asymptotic frequencies due to the parameter $\delta$. The average phase coherence of the neural oscillators is not affected either by the inclusion of activity dependence, as shown in Fig. 10d. Therefore, we find that the spreading dynamics is affected by its dependence on neuronal dynamics, while the neuronal dynamics themselves appear mostly unaffected.

## Discussion

Reduction to slow manifolds and ad hoc averaging allows us to elucidate the heterodimer-oscillator dynamics in the singular limit. The heterodimer-oscillator operates in two regimes, as the oscillators are either phase-locked or drifting. In the phase-locking regime, the heterodimer-oscillator exhibits dynamics similar to the original heterodimer model, whereas in the drifting regime, the dynamics is similar to the skewed heterodimer model. This is not surprising, as the links are symmetric during the phase-locking regime and asymmetric during the drifting regime. In both regimes, we identify a pair of healthy and toxic fixed points exchanging stability at a transcritical bifurcation (for small $\delta$ in the drifting regime). Inspecting the evolution equations for the heterodimer-oscillator, we see that it inherits the symmetric fixed points of the heterodimer model when $A_1=A_2$ (under phase-locking), as the transport terms cancel each other out. Additionally, there is a unique healthy fixed point in both the drifting and phase-locking regimes; setting $v_1 = v_2 = 0$ reduces the equilibrium conditions to a determinate system of linear equations. The healthy fixed point only exists in the phase-locking regime when $K \le |\Delta \omega |$, and likewise, the healthy fixed point only exists in the drifting regime $K \ge |\Delta \omega |$. However, the nonlinearities introduced by the heterodimer-oscillator may introduce novel (toxic) equilibria beyond those identified herein. We also established—when healthy species *u* are close to their steady-state values—the direction of the vector field of $\Delta v$ at the border between the phase-locking and drifting regimes. In doing so, we ruled out the possibility of stable limit cycles crossing the regime border at the singular limit. However, we cannot rule out the existence of limit cycles within either of the regimes. Nonetheless, it seems likely that the heterodimer-oscillator approaches the phase-locking regime for $K \ge |\Delta \omega |$ and the drifting regime for $K < |\Delta \omega |$.

The large timescale separation one typically has for the evolution of neurodegenerative diseases justifies the analysis of the singular limit. While smaller timescale separation may alter the dynamics of the system, the numerically obtained solution trajectories shown in Fig. 4 suggest that the heterodimer-oscillator is well-approximated by the singular-limit dynamics even for $\epsilon \approx 0.1$. In particular, numerical simulations indicate that the stability of the phase-locking and drifting regimes is accurately described by the singular limit analysis, even for complex networks. However, we cannot rule out the occurrence of more complex dynamical phenomena for larger $\epsilon$, though it has not yet been observed.

In addition to a smaller timescale separation, generalizations of the oscillator dynamics and the coupling between oscillator and spreading dynamics are likely to induce new dynamical phenomena. We chose a simple relationship between the heterodimer dynamics and the oscillator intrinsic frequencies by setting $\widehat{\omega }(v) = \omega - c v$, which can be interpreted as a first-order Taylor expansion of a more complicated $\widehat{\omega }(v)$. From the expansion of the toxic fixed point in the drifting regime in the singular limit, we have that $|c\Delta v| = |\Delta \widehat{\omega } - \Delta \omega | = \mathcal {O}(\sqrt{\Delta \omega ^2 - K^2})$ which implies that $\Delta \widehat{\omega } \rightarrow \Delta \omega$ as $K \rightarrow |\Delta \omega |$. In other words, the heterodimer-oscillator SNIC bifurcation at $K = |\Delta \widehat{\omega }|$ becomes indistinguishable from $K = |\Delta \omega |$. As such, the Kuramoto oscillators appear to only depend on the oscillator parameters. As such, higher-order terms in $\widehat{\omega }(v)$ may be necessary to induce qualitative changes in Kuramoto dynamics. The activity-dependence, $A(\theta )=\epsilon \dot{\theta }$, was also assumed to be linear, though this relationship may be more complicated in reality. In the case of neurodegenerative disease, more empirical data is necessary to determine the quantitative relationship between neuronal firing and protein transport rate. A smaller timescale separation may also lead to more interesting behavior in the Kuramoto dynamics. If the temporal changes of the effective intrinsic frequencies $\widehat{\omega }$ are faster, there may be transient chimera-like states, where parts of the network are severely affected by the spreading and others are not, leading to clusters synchronized at different frequencies. Another interesting scenario involves several heterodimer species on the same network. These species may interact (Thompson et al. 2020) and even affect the underlying oscillator dynamics differently (Alexandersen et al. 2023). The latter case could be realized by having two heterodimer species evolving, where one speeds up (negative *c*) and the other slows down (positive *c*) the intrinsic frequencies of the oscillators. It is also possible to modify the heterodimer-oscillator to affect the coupling strengths between oscillators as opposed to their intrinsic frequencies. With the addition of coupling strength adaptation rules, we expect more elaborate oscillator dynamics in the symmetry-breaking (drifting) regime in line with previous research (Jüttner and Martens 2023).

We have focused on oscillatory processes that accelerate spreading ($\delta > 0$) and spreading processes that slow oscillatory processes ($c>0$). Nonetheless, the heterodimer-oscillator may be fit for modeling phenomena where either *c* or $\delta$ are negative. By asserting a lower bound on $\delta$, we can extend the linear stability results to $c,\delta \in \mathbb {R}$. However, we cannot repeat the regime border analysis when both $c < 0$ and $\delta <0$. In this case, one can imagine a positive feedback loop leading to a stable toxic solution for large $\Delta v$, independently of the stability of the healthy fixed point; the node with the highest toxic concentration will increase in activity, causing a reduction in outward transport, followed by an increase in toxic concentration and so on. Still, we are yet to identify applications to which both *c* and $\delta$ should be negative.

From both the discussion on the toxic equilibrium stability and the network simulations, we conclude that there are two modes of toxic propagation in the heterodimer-oscillator: conversion-dominated spreading and shunting-dominated spreading. The latter does not exist in the heterodimer model, in which a redistribution of healthy proteins precedes the production of toxic proteins. When the healthy proteins are distributed through the network according to the activity gradient, some nodes are overwhelmed by the amount of healthy protein, and thus, the conversion to toxic proteins proceeds. In conversion-dominated spreading, toxic proteins are produced whether or not there is an activity gradient. The activity gradient causes some nodes to have more protein than others, but it is not the determining factor causing an outbreak. Shunting-dominated spreading occurs when the parameters are close to the transcritical bifurcation, whereas conversion-dominated spreading occurs when the parameters are far away from the transcritical bifurcation. In Alzheimer’s disease, it has been hypothesized that lowered toxic clearance may initiate the disease. Therefore, the heterodimer-oscillator model posits that the disease initiates with shunting-dominated spreading and that the gradient of neuronal activity levels determines which regions are first affected.

The formulation of the heterodimer-oscillator was primarily motivated by the case of Alzheimer’s disease and other neurodegenerative diseases. The impact of neuronal activity on pathological protein spreading patterns is becoming increasingly clear and provokes the need for mechanistic, mathematical modeling of the bidirectional relationship between disease progression and neuronal activity. Building our model from mechanistic principles from the neuroscientific literature, we provide a simple mathematical model of this relationship. Importantly, the heterodimer-oscillator provides falsifiable hypotheses on the nature of prion-like spreading; protein spreading patterns follow a neuronal activity gradient and more extreme gradients push the brain towards a pathological state. We have also demonstrated that the heterodimer-oscillator indeed alters the tau staging patterns when simulated on a human brain connectome. It is not uncommon for patients to deviate from the stereotypical Braak staging patterns, and the heterodimer-oscillator may thus provide a mechanistic explanation for such aberrations. Future work is needed to establish the predictive power and ramifications of the heterodimer-oscillator in applications to neurodegenerative disease modeling.

## Data Availability

Supporting data for this research will be given upon request.

## Coefficients of the cubic

The toxic solution for the skewed heterodimer model is given by the real positive solution (when it exists) of a cubic equation for the toxic fixed point ($v_2\not =0$) given by

$$\begin{aligned} c_0+c_1 v_2+c_2 v_2^2+c_3 v_2^3=0 \end{aligned}$$

with

$$\begin{aligned} c_0=&\, (A + \ell ) (k_3 (A + 2 \ell + k_3) + k_{1}^{4} + 4 \ell (A + \ell ) k_{0}^{2} k_{2}^{2} - 2 (A + 2 \ell ) k_{0} k_{1} k_{2}\\&\times (A^{2} + 2 \ell (\ell + k_3) + A (2 \ell + k_3) - k_{0} k_{2}) + k_{1}^{3} (2 (A + 2 \ell ) k_3 (A + 2 \ell + k_3)\\&- (A + 2 (\ell + k_3)) k_{0} k_{2}) + k_{1}^{2} ((A + 2 \ell )^{2} k_3 (A + 2 \ell + k_3)c - (3 A^{2} + 8 \ell (\ell + k_3) \\&+ 4 A (2 \ell + k_3)) k_{0} k_{2} + k_{0}^{2} k_{2}^{2})),\\ c_1=&\, k_{2} (A^{4} k_{1} k_3 + A^{3} (3 k_{1}^{2} k_3 - 2 k_{0} k_{1} k_{2} + 2 k_{0} k_3 k_{2} + 6 k_{1} k_3\ell ) + A^{2} (2 k_{1}^{3} k_3 \\&+ 2 k_{0} k_{2} (k_3^{2} + 2 k_{0} k_{2} + k_3 \ell ) + k_{1}^{2} (3 k_3^{2} - k_{0} k_{2} + 15 k_3 \ell )\\&- k_{1} (k_3^{3} + 6 k_{0} k_3 k_{2} + 8 k_{0} k_{2} \ell - 14 k_3 \ell ^{2})) + (k_{1} + 2 \ell ) (k_{1}^{2} k_3 (k_3^{2} + 5 k_3 \ell + 6 \ell ^{2})\\&+ k_{0} k_{2} (-2 k_3 \ell ^{2} + k_{0} k_{2} (k_3 + 2 \ell )) + k_{1} (2 k_3 \ell ^{2} (k_3 + 2 \ell ) - k_{0} k_{2} (2 k_3^{2} + 7 k_3 \ell + 4 \ell ^{2})))\\&+ A (k_{1}^{3} k_3 (3 k_3 + 7 \ell ) + 2 k_{0} k_{2} (k_3 (k_3 - 2 \ell ) \ell + k_{0} k_{2} (k_3 + 4 l)) \\&+ k_{1}^{2} (-5 k_{0} k_{2} (k_3 + l) + k_3 \ell (11 k_3 + 26\ell )) + 2 k_{1} (k_{0}^{2} k_{2}^{2} - k_{0} k_{2} (k_3^{2} + 10 k_3\ell + 7\ell ^{2})\\&+ k_3\ell (-k_3^{2} + k_3\ell + 8\ell ^{2}))),\\ c_2=&\, k_{2}^{2} (A^{3} (k_{1} - k_3) k_3 + A^{2} k_3 (k_{1}^{2} + 2 k_{1} k_3 - k_3^{2} - 4 k_{0} k_{2} + 5 k_{1}\ell - 3 k_3\ell )\\&+ (k_3 + 2\ell ) (-3 k_{0} k_3 k_{2}\ell - k_{0} k_{1} k_{2} (2 k_3 +\ell ) + k_{1}^{2} k_3 (2 k_3 + 3\ell ) + k_{1} k_3\ell (3 k_3 + 4\ell )) \\&+ A (k_{1}^{2} k_3 (3 k_3 + 5\ell ) + k_{1} (k_3^{3} - 3 k_{0} k_3 k_{2} + 8 k_3^{2}\ell - 2 k_{0} k_{2}\ell \\&+ 10 k_3\ell ^{2}) - k_3 (k_3\ell (k_3 + 2\ell ) + k_{0} k_{2} (3 k_3 + 10\ell ))),\\ c_3=&\,k_3 k_{2}^{3} (A + k_3 + 2\ell ) (A k_3 + k_3\ell + k_{1} (k_3 +\ell )). \end{aligned}$$

## Critical clearance for the skewed heterodimer model

In this section, we show that the critical clearance of the 2-node skewed heterodimer model is constrained to the interval $[k_0 k_2/k_1, 2k_0 k_2 / k_1]$ and is monotonically increasing in the activity parameter *A*.

### Critical clearance bounds

In the skewed heterodimer 2-node system (Sect. 2.2), the healthy fixed point switches stability at a critical value for toxic clearance given by

37 $$\begin{aligned} k_3^{\text {crit}} = \frac{k_0 k_2 + \kappa ^{\text {crit}}}{k_1}, \end{aligned}$$

where $\kappa ^{\text {crit}}$ is given in Sect. 2.2. We now verify the statement that $\frac{k_0 k_2}{k_1} \le k_3^{\text {crit}} \le 2 \frac{k_0 k_2}{k_1}$ by showing that $0 \le \kappa ^{\text {crit}} \le k_0 k_2$. As all parameters are nonnegative the following inequality holds

38 $$\begin{aligned} &  4 k_0 k_2 \left( k_1 \left( A+k_1+2 l\right) \left( k_1 (A+2 l)+4 l (A+l)\right) +k_0 k_2 \left( k_1+2 l\right) \left( 2 (A+l)+k_1\right) \right) \nonumber \\ &  \quad \ge 0. \end{aligned}$$

The inequality can be rewritten in terms of $s_0$ and $s_1$ as defined in Sect. 2.2, giving

39 $$\begin{aligned} (2 l + A + k_1)^2(k_1(2l + A)+2k_0 k_2)^2 \ge s_0^2 + s_1. \end{aligned}$$

Taking the square root of both sides and rearranging gives us the desired $\kappa ^{\text {crit}} \le k_0 k_2$. As mentioned in Sect. 2.2, $\kappa ^{\text {crit}} \ge 0$ since all parameters are nonnegative. Conclusively, we have that $\frac{k_0 k_2}{k_1} \le k_3^{\text {crit}} \le 2 \frac{k_0 k_2}{k_1}$.

### Monotonic dependence of critical clearance on activity

We now verify the statement that $k_3^{\text {crit}}$ is monotonically increasing in *A* by showing that $\frac{\delta \kappa ^{\text {crit}}}{\delta A} \ge 0$. As all parameters are nonnegative, the following inequality holds

40 $$\begin{aligned} \frac{4 A k_0 k_2}{k_1^2 (2 l + A + k_1)^4}[B_1 + B_2 + B_3 + B_4] \ge 0, \end{aligned}$$

where

41a $$\begin{aligned} B_1&= k_1^3 \left( A+k_1+2 l\right) ^4 \left( k_1 (A+4 l)+4 l (A+2 l)\right) , \end{aligned}$$

41b $$\begin{aligned} B_2&= k_0 k_1^2 k_2 \left( k_1+2 l\right) \left( A+k_1+2 l\right) ^2 \left( 4 A^2+k_1 (5 A+4 l)+14 A l+8 l^2\right) , \end{aligned}$$

41c $$\begin{aligned} B_3&= 4 A k_0^2 k_1 k_2^2 \left( k_1+2 l\right) \left( A+k_1+2 l\right) \left( A+2 k_1+4 l\right) , \end{aligned}$$

41d $$\begin{aligned} B_4&= 4 A k_0^3 k_2^3 \left( k_1+2 l\right) ^2. \end{aligned}$$

Rearranging the above inequality gives

42 $$\begin{aligned} &  \left( \frac{4 A k_0^2 k_2^2 \left( k_1+2 l\right) }{k_1 \left( A+k_1+2 l\right) ^2}+\frac{2 A k_0 k_2 \left( A+2 k_1+4 l\right) }{A+k_1+2 l}+k_1 (A+2 l) \left( A+k_1+2 l\right) \right) ^2\nonumber \\ &  \quad \ge s_0^2 + s_1, \end{aligned}$$

where $s_0$ and $s_1$ are again defined as in Sect. 2.2. Taking the square root of the right- and left-hand side of the above inequality, and dividing both sides by $4 (2\,l + A + k_1)^2 \sqrt{s_0^2 + s_1}$ produces

43 $$\begin{aligned} k_1 \frac{\partial \kappa ^{\text {crit}}}{\partial A} \ge 0, \end{aligned}$$

showing that $k_3^{\text {crit}}$ is monotonically increasing in *A*. The full expression of the partial derivative has been omitted due to its length.
